# Clinical characteristics and molecular mechanisms underlying bladder cancer in individuals with spinal cord injury: a systematic review

**DOI:** 10.1186/s12894-024-01457-0

**Published:** 2024-05-22

**Authors:** Xin-Lei Wang, Yi-Xuan Wang, Jun-Zhi Chen, Xin-Yu Liu, Xing Liu, Qi-Kai Zhong, Zi-Lin Zhao, Zhen-Duo Shi, Cong-Hui Han

**Affiliations:** 1 Department of Urology, Xuzhou Clinical School of Xuzhou Medical University, Xuzhou Central Hospital, Xuzhou, Jiangsu China; 2 Suzhou High School of Jiangsu Province, Suzhou, Jiangsu China; 3 Department of Urology, Xuzhou Central Hospital, Jiefang South Road, No. 199, Xuzhou, Jiangsu China; 4 School of Life Sciences, Jiangsu Normal University, Xuzhou, Jiangsu China; 5 Jiangsu Provincial Engineering Research Center of Cancer Cell Therapy and Translational Medicine, Xuzhou City Engineering Research Center of Cancer Cell Therapy and Translational Medicine, Jiangsu, China

**Keywords:** Spinal cord injury, Bladder cancer, Clinical characteristics, Molecular mechanisms, NO, miRNA

## Abstract

**Background:**

Patients with spinal cord injury have a relatively high risk for bladder cancer and often complicated with bladder cancer in advanced stages, and the degree of aggressiveness of malignancy is high. Most of the literature is based on disease clinical features while, our study reviews the clinical characteristics and molecular mechanisms of spinal cord injury patients with bladder cancer, so that it might help clinicians better recognize and manage these patients.

**Method:**

We searched PubMed, Web of Science and Embase, using retrieval type like (“Neurogenic Lower Urinary Tract Dysfunction” OR “Spinal cord injury” OR “Spinal Cord Trauma”) AND (“bladder cancer” OR “bladder neoplasm” OR “bladder carcinoma” OR “Urinary Bladder Neoplasms” OR “Bladder Tumor”). In Web of Science, the retrieval type was searched as “Topic”, and in PubMed and Embase, as “All Field”. The methodological quality of eligible studies and their risk of bias were assessed using the Newcastle-Ottawa scale. This article is registered in PROSPERO with the CBD number: CRD42024508514.

**Result:**

In WOS, we searched 219 related papers, in PubMed, 122 and in Embase, 363. Thus, a total of 254 articles were included after passing the screening, within a time range between 1960 and 2023. A comprehensive analysis of the data showed that the mortality and incidence rates of bladder cancer in spinal cord injury patients were higher than that of the general population, and the most frequent pathological type was squamous cell carcinoma. In parallel to long-term urinary tract infection and indwelling catheterization, the role of molecules such as NO, MiR 1949 and Rb 1. was found to be crucial pathogenetically.

**Conclusion:**

This review highlights the risk of bladder cancer in SCI patients, comprehensively addressing the clinical characteristics and related molecular mechanisms. However, given that there are few studies on the molecular mechanisms of bladder cancer in spinal cord injury, further research is needed to expand the understanding of the disease.

**Supplementary Information:**

The online version contains supplementary material available at 10.1186/s12894-024-01457-0.

## Introduction

Bladder cancer (BC) is a common malignancy within the field of urology. According to GLOBOCAN’s 2020 estimates, there were 573,278 newly diagnosed cases of BC and 212,536 deaths caused by this disease [1]. While, in the 2022 cancer statistics, 81,180 new BCs, and 17,100 BC deaths were projected in the United States [2]. Urothelial carcinoma (UC) comprises Approximately 70% of all tissue, while, non-urothelial carcinomas such as squamous cell carcinoma (SCC), adenocarcinoma, and neuroendocrine tumor contribute to the remaining proportion [3]. Squamous cell carcinoma (SCC) is the most prevalent among individuals with spinal cord injury (SCI) who have BC, accounting for approximately 36.8% of reported cases [4].

Recent studies have revealed that SCI affects approximately 25–30 million individuals globally [5]. According to the 2016 Global Burden of Diseases, Injuries, and Risk Factors (GBD) report, there is an approximate annual incidence of nearly one million new cases of SCI [6]. Individuals suffering from SCI frequently experience complications, including problems with urination, urinary tract infections, and the need for long-term catheter use. These complications can eventually result in BC, typically occurring 15–20 years after the onset of the disease. This type of cancer is often characterized by bladder muscle infiltrating squamous cell carcinoma, significantly affecting the prognosis of patients [7, 8]. Numerous researchers have examined the macroscopic elements of SCI and BC and found that individuals with SCI exhibit a higher degree of malignancy [9] and are diagnosed with BC 1–20 years before the general population [5].

After searching the literature, we noted that most research on individuals with SCI and BC focuses on the clinical aspects. While fewer studies investigate the underlying mechanisms of BC development. Therefore, it would be recommended to link the clinical parameters and molecular mechanisms to delineate the potential connection between the macro and micro pathogenesis levels. This article provides a comprehensive overview of the clinical characteristics and molecular mechanisms associated with SCI and BC, with the aim to aid clinicians in enhancing understanding and management of patients affected by this condition.

## Methods

The authors searched for relevant literature by conducting searches in databases such as PubMed and Web of Science, using retrieval type like (“Neurogenic Lower Urinary Tract Dysfunction” OR “Spinal cord injury” OR “Spinal Cord Trauma”) AND (“bladder cancer” OR “bladder neoplasm” OR “bladder carcinoma” OR “Urinary Bladder Neoplasms” OR “Bladder Tumor”). In Web of Science, the retrieval type was searched as “Topic”, and in Pubmed and Embase, as “All Field”. Subsequently, we evaluated the titles and abstracts of the literature, carefully the ones that were most pertinent to the study. This screening method includes not only the clinical type of articles, but also the required basic research. They then examined the references cited in these selected works. Study reporting adheres to PRISMA guidelines. And this article is registered in PROSPERO with the CBD number: CRD42024508514.

### Study eligibility

Study selection criteria included reporting age of onset, incidence of bladder cancer, English literature and Based on the title and abstract, were included in ref. While, Exclusion criteria were non-English publications, studies with insufficient or unconfirmed information, or when patients had bladder cancer before the SCI.

According to the criteria of the PRISMA Guidelines, the questions were screened to evaluate whether they met the inclusion requirements and to reevaluate the selected studies.

### Data extraction and quality assessment

Data were collected independently and no automated tools were used. Whenever problems were encountered, senior expert (Corresponding Author) were consulted. The age of onset, incidence, number of deaths and pathological type of bladder cancer in SCI patients mentioned in the included literature were introduced into Excel. The risk of bias and methodological quality of eligible studies were assessed using the Newcastle-Ottawa scale, which involved eight items in the area of choice, comparability and outcome of cohort studies. The three items were evaluated and scored separately (either 1 or 2 points). In this analysis, studies with NOS scores of 1–3,4–6, and 7–9 were defined as low, moderate, and high-quality studies, respectively. Poor-quality studies were not excluded from this review.

## Results

**Fig. 1:**
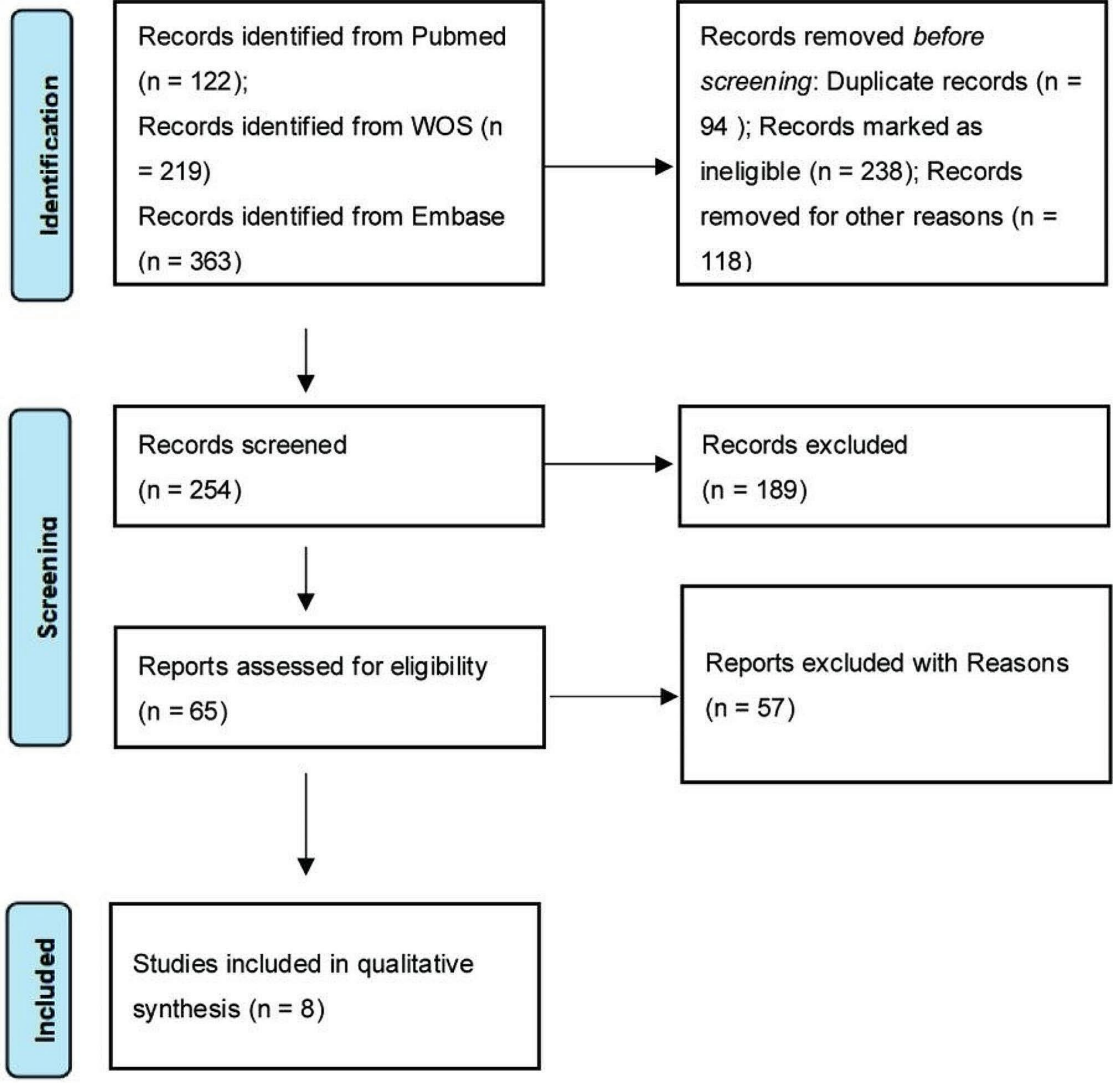
PRISMA flow diagram

Using the above search criteria, a total of 704 documents were considered. After filtering out duplicates 254 documents remained and After screening by title, abstract and full text, 246 literatures were excluded for various reasons. Finally, A total of 8 papers were included in this review **(**Fig. 1**)**.

Among the included studies, seven were of medium to high quality, and two were of low quality. The specific scoring details are shown in Table 1. This study used the the Newcastle-Ottawa scale for the quality assessment.

**Table 1:** The score chart of NOS

Year	Author	Slelection				Comparability	Outcome			Points		Quality of Studies	
		1	2	3	4	5	6	7	8				
2015	Laura S	*	*	*	*	*	*	*	*		8		High
2022	Floriane M	*	*	*	*	**	*	*	—		8		High
2020	Ralf Böthig	*	—	*	*	*	*	*	—		6		High
2017	Gui-Zhong, L	—	—	*	*	—	*	*	—		4		Intermediate
2015	Ho, C.H	*	*	*	*	**	*	*	*		9		High
2021	Ralf Böthig	*	*	*	*	**	*	*	*		9		High
2021	Ammirati Enrico	*	—	—	—	—	*	—	—		2		Low
2023	Hidalgo Romero A	*	—	—	—	—	*	—	—		2		Low

### Characteristics observed in the clinical setting

#### Morbidity and mortality

A retrospective analysis was performed by Floriane et al. on French data from 2010 to 2018 [10]. To assess the incidence of bladder cancer (BC) in patients with multiple sclerosis (MS), spinal cord injury (SCI), and spina bifida (SB). This study included a total of 50,243,847 individuals, among whom 2015 patients (0.004%) were identified as having BC in conjunction with neurogenic bladder. With average age of onset 65.4 ± 12.3 years and affecting more men than women. And also showed that SCI patients had a higher incidence of BC than MS and SB, with a rate of 791.1/100 000 persons/year.

Similar findings were obtained by Böthig et al. [11]. who conducted a retrospective analysis of 7004 individuals suffering from SCI between 1998 and 2018. Four patients (incidence approximately 0.0571%) were diagnosed with BC after several years, with an average age of onset of 65.5 years (median age of 64 years [52–82]). In addition, Bothig et al. [12] found that the age at diagnosis of bladder cancer was 56.6, and the age of initial diagnosis is younger, at 19 years old. The average time spent in patients with urinary catheters from spinal cord injury to bladder cancer was significantly longer than that spent in patients with no-urinary catheters. Noteworthy, the age of onset and incidence rates were similar in both studies, and this finding is consistent with previous studies **(**Table 2**)**.

**Table 2:** The occurrence of BC among individuals who have experienced a SCI

Author and Title	Study period(year)	Country	The mean age of onset (years)	incidence of BCa
Laura S.et al.	1960–2009	USA	48–61	N/A
Floriane M.et al.	2010–2018	France	65.4 ± 12.3	791.1 ± 170.5/100,000 patients/year
Ralf Böthig. et al.	1998–2018	Germany	65.5(median 64, range 52–82)	0.0571%
Gui-Zhong, L.et al.	1966–2010	N/A	50(95% CI, range 45–55)	0.601%
Ho, C.H.et al.	1998–2002	China Taiwan	47.42 ± 15.84	0.551% (10/1816)
Ralf Böthig. et al.	2012–2019	Germany, Austria, Switzerland	56.6(range 19–88)	N/A
Ammirati Enrico et al.	2011–2020	Italy	N/A	9.09% (1/11)
Hidalgo Romero A	2007–2023	Netherlands	N/A	N/A*

N/A: not available; SCI: spinal cord injury; BC: bladder cancer

However, other authors have derived incidence rates that are about ten times higher than the above two. Gui-Zhong et al. used the MEDLINE, EMBASE, CINAHL and the Cochrane Register database to search the literature found that out of 99,237 individuals with SCI, 596 were diagnosed with BC [4]. (An occurrence rate of 0.601%), with an average age of onset around 50 years. While, The incidence of bladder SCC was higher than that in the general population. To find the effect of chronic indwelling catheterization on the occurrence of bladder cancer, a study compared the effect of chronic indwelling urinary catheters(CIDC) between a non-SCI group and an SCI-group (control group) of patients. The incidence of bladder cancer in the SCI group and the CIDC-non-SCI group was 68.90 and 102.53 per 100,000 person-years and both were higher than the control group (12 per 100,000 person-years). This study was conducted in Taiwan on 1,816 individuals with SCI. Among who, 10 were diagnosed with BC, (incidence rate of 0.551%) with average age of onset of BC 47.42 ± 15.84 years [13]. Ammirati Enrico [14] evaluated 11 patients with SCI from 2011 to 2020 and all patients were treated with CIC. At late follow-up, one patient had found high-grade non-invasive bladder cancer after radical cystectomy. In this study, the investigators only randomly selected 11 SCI patients and did not include all SCI patients, so the results were not universal. However, the results show that long-term urinary catheterization treatment may increase the risk of bladder cancer. Hidalgo Romero A used a similar approach [15]. The authors selected 10 patients who developed bladder cancer after SCI / NTD from 2007 to 2023. By collecting various clinical and pathological information of patients, it was found that all patients had high-grade non-urothelial cell carcinoma. Three cases developed lymph node metastases. This follows that bladder cancer caused by SCI may have a worse prognosis.

The incidence of bladder cancer found in the above studies is not exactly the same. And The differences may be related to the diseased race, living environment and habits. Despite the differing conclusions among experts, all studies show that individuals with SCI can potentially experience BC at a younger age than the general population**(**Table 2**)**.

A study conducted between 1960 and 2009 by Laura et al. involving 45,486 patients with SCI and a total follow-up of 566,532 person-years, revealed that out of the 10,575 patients who passed away, 99 deaths were attributed to BC [16]. The standardized mortality ratio (SMR) for BC was significantly higher (6.7) in SCI patients compared to the general population, indicating a substantial difference. Likewise, Böthig et al. yielded comparable outcomes. In a study conducted in Germany between 1998 and 2014 involving 6995 patients with SCI [17]. Among the cohort of 24 individuals diagnosed with spinal cord injury (SCI) and bladder cancer, a total of 12 individuals succumbed to the disease (50%). The median survival time for this group was found to be 11.5 months, while the mean survival time was calculated to be 22.71 months, with a standard deviation of 30.42 months **(**Table 3**)**. Both studies discovered that individuals with SCI succumbed to BC at a younger age than the overall population. Additionally, they observed a correlation between SCI patients and a lower pathological staging, indicating that SCI patients tend to develop BC with a less favorable prognosis. These findings are consistent with the prevailing body of published literature on the subject matter.

**Table 3:** The death rate of BC among individuals who have experienced SCI

Author and Title	bladder cancer cases(number)	Number of deaths	Median survival(months)	pathology	T category	
Laura S.et al.	N/A	99	N/A	N/A	N/A	
Ralf Böthig. et al.	4	0	N/A	TCC	3×pTa 1×pT1	
Ralf Böthig. et al.	24	12	11.5 (mean 22.71, standard deviation 30.42)	19×TCC、4×SCC、 1× Complete undifferentiated cancer	19×≥T2、2×T1 3×pTa	

N/A: not available; SCI: spinal cord injury; BC: bladder cancer; TCC: Transitional cell carcinoma; SCC: squamous cell carcinoma

#### Mechanism of occurrence

**Fig. 2:**
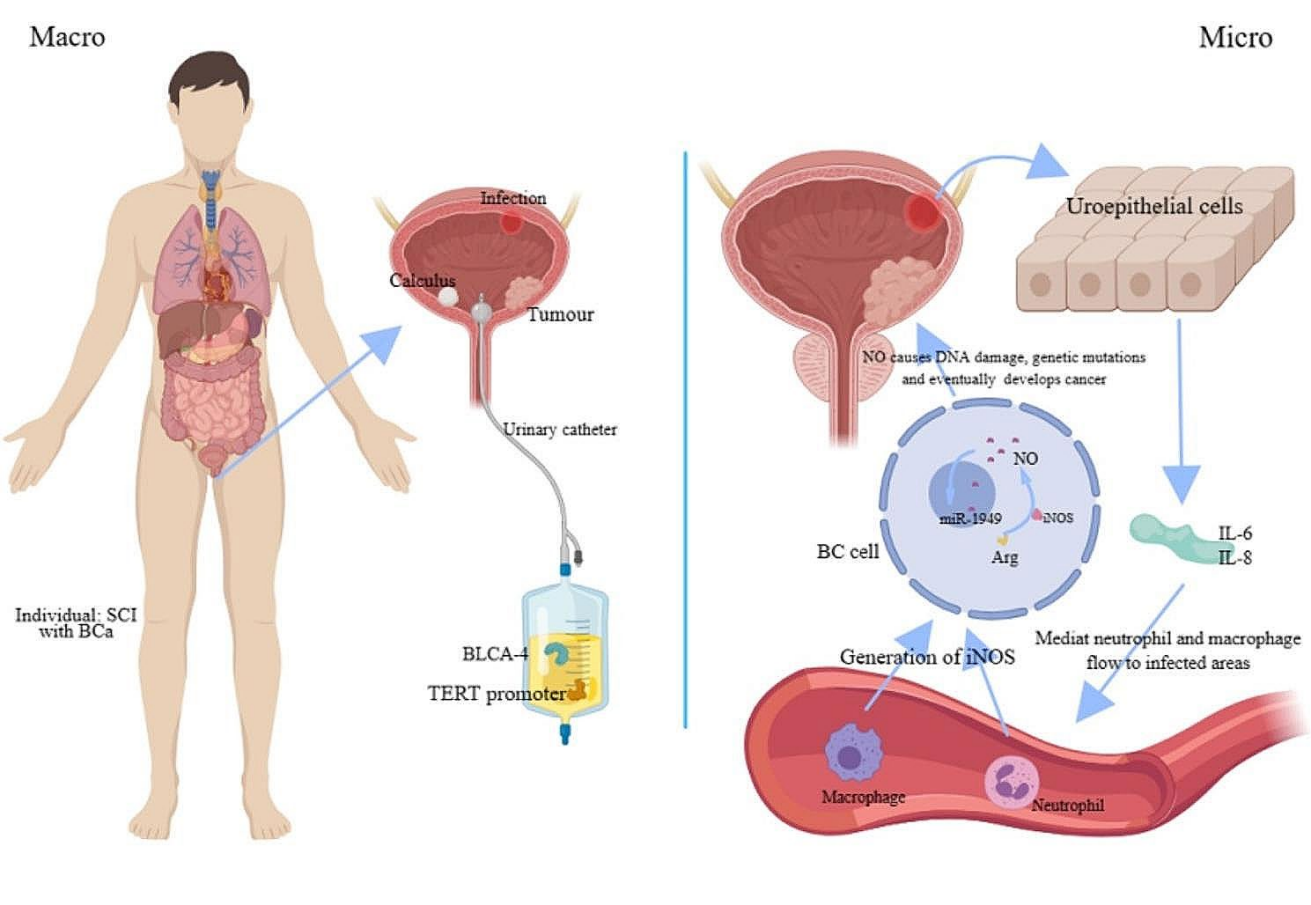
A sketch of the pathogenesis: bladder cancer in patients with spinal cord injury

BC is primarily caused by smoking and prolonged exposure to chemicals such as aromatic amines [18]. While, diet may also increase the occurrence of bladder cancer, as Aveta et al. found that “red meat” and “processed meat” may be positively associated with bladder cancer risk [19]. In individuals with neurogenic bladder resulting from SCI, healthcare facilities often use catheters or suprapubic cystostomies for extended durations, which, along with the factors mentioned above, have been linked to chronic bladder inflammation and an increased risk of BC [20, 21]. Furthermore, the prolonged use of catheters can lead to intricate urinary tract infections and the formation of bladder stones, increasing the probability of developing BC **(**Fig. 2**)** [22]. Studies have found that lower bladder compliance and higher maximum bladder pressure are observed in SCI mice. Overactive bladder(OAB) increases, and an indwelling catheter is needed to relieve the patient’s disease [23]. For OAB, studies have found that capsaicin-sensitive bladder afferent neurons (B-AN) and type A voltage-gated potassium channel (K_A_) function were decreased in SCI mice compared with the control group [24]. Indicating that reduced K_A_ channel activity is associated with hyperexcitability of capsaicin-sensitive C fiber B-AN after SCI. Therefore, studies targeting neuronal-type K_A_ ion channels are expected to be a new therapeutic target for OAB. This method may be used to reduce the possibility of long-term indwelling urinary catheters. Von Siebenthal et al. [25] compared bladder function in the SCI (neurogenic) and bladder outlet obstruction (obstructive) mice by repeated urodynamic examination. This study found that the SCI group had dysfunctional bladder detrusor and sphincter and significantly increased bladder pressure 1 week after injury, and the bladder gradually developed fibrosis throughout the process. These factors are closely related to SCC and muscle infiltration into the bladder in cases of BC. However, what is the link between the two conditions at a microscopic level? Temporal and spatial mutations were discovered in the telomerase reverse transcriptase (TERT) promoter of BC tissue from the general population and in keratinized/non-keratinized squamous epithelial tissue from patients with neurogenic bladder, as found by Taylor et al. [26]. Understanding why chronic cystitis caused by indwelling catheters is related to a higher susceptibility to BC can be facilitated by considering the proposal that this mutation could serve as a plausible pathway for the progression of benign lesions to BC.

Nevertheless, a Meta-analysis revealed that approximately 1% of individuals with SCI who underwent prolonged catheterization experienced the development of BC [27]. Whilst, more than 50% of SCI patients without indwelling catheters experienced the development of BC. Meanwhile, Kalisvaart et al. found that 50% of the individuals included in their research who had BC were not using a catheter for an extended period. BC in patients with SCI can be attributed to various factors [28]. In line with the above data, it is reasonable to infer that indwelling catheters, persistent bladder inflammation, and urinary tract infections are the primary culprits, however, it is important to maintain a skeptical mindset and further investigate the diverse pathways of BC development in SCI patients.

#### Screening and diagnosis

The topic of contention among medical professionals revolves around the frequency of bladder cancer screening, the appropriate screening methods, and the efficacy of early screening in detecting bladder cancer in SCI patients [29]. According to Laura et al. [16], the average duration between SCI and the identification of BC in patients ranged from 16 to 34 years. A study conducted in Taiwan, in a group of 54,401 individuals with SCI monitored for an average of six years, results did not exhibit a greater likelihood of being diagnosed with BC compared to those without SCI [30]. Consequently, it is advisable to enhance the frequency of patient evaluations for individuals with SCI beyond a decade of SCI to attain prompt identification and medical intervention. and some researhers suggest conducting annual cystoscopy and urine cytology [29].

Urine cytology is not recommended for routine urinalysis in this group of patients due to the possibility of haematuria and pyuria caused by an indwelling catheter. Davies et al. have shown that urine cytology or urine biomarkers do not possess sufficient reliability as a screening technique for individuals with spinal cord injury. A study conducted between 1999 and 2004 examined 457 patients with SCI to evaluate the efficacy of urine biomarkers, specifically the BTA stat test, survivin assay, and cytology, in detecting bladder cancer cases. The findings of this study indicated that these aforementioned methods were not effective screening tools for bladder cancer [31]. Kinde et al. found that approximately 66% of patients with muscle-invasive BC carried TERT promoter activating mutations, which can be detected in urine and are strongly associated with BC recurrence, suggesting that TERT promoter mutations could serve as a useful marker for early detection and monitoring of BC **(**Fig. 2**)** [32]. Regarding cystoscopy, some scholars believe that it is still the ultimate basis for diagnosis [33]. In addition to the above mentioned techniques, alternative approaches are currently available to identify BC in individuals with SCI.

Konety [34] measured the urinary nuclear matrix protein BLCA-4 in the urine of healthy individuals, patients diagnosed with BC, and patients with SCI. The study revealed that 53 of 55 (96.36%) BC patients and 38 of 202 (18.81%) SCI patients had BLCA-4 levels that exceeded the defined limit **(**Fig. 2**)**. Despite the absence of a connection between increased BLCA-4 levels and indwelling catheters, chronic bladder inflammation, and urinary tract infections, the presence of BLCA-4 in the urine holds great diagnostic promise. And, hopefully, it could serve as a valuable method for the early identification of BC in individuals with SCI.

In summary, various screening techniques exist for individuals with SCI in conjunction with BC, although a standard and universally recognized protocol is yet to be established. The presence of hematuria, back pain resulting from ureteral obstruction, recurrent urinary tract infections, or the identification of a pelvic mass during physical examination in individuals with SCI signifies a significant risk factor for bladder cancer. Consequently, it is imperative that these patients undergo routine screening procedures.

### Molecular mechanisms

#### Nitric oxide (NO)

The human body relies on NO for its vital biological functions, serving as neurotransmitter and other transmitters. For example, NO plays the role of messenger molecule, and when vascular pressure increases, it increases NO release by activating Ca + in endothelial cells, and then plays the role of dilating blood vessels [35]. NO also can act as a retrograde neurotransmitter. Glutamate activates N-methyl-d-aspartate receptor (NMDAR) to import Ca 2 + into the cell, and then generates nitric oxide. This is a reverse neurotransmitter that maintains glutamate secretion at the presynaptic terminals [36].

It contributes to vasodilation and prevention of platelet aggregation by enhancing guanylate-activating enzyme activity, which results in elevated levels of cyclic-geranyl phosphate (cGMP) [37]. Furthermore, NO has a significant impact on inflammation and cancer. Urinary tract infections cause the secretion of IL-6 and IL-8 by urinary tract epithelial cells, facilitating the migration of neutrophils to the infected region. Frequent urinary bladder inflammation, which occurs due to the presence of catheters in patients with SCI, is commonly accompanied by infiltration of cells that cause inflammation. Macrophages and neutrophils in inflammatory tissues have been found to produce inducible NO synthase (iNOS) (Fig. 2) [38]. Gecit et al. [39] found that the concentrations of harmful substances malondialdehyde, NO, and prolyl peptidase were elevated in cancerous tissues compared to non-tumor tissues. While, In contrast, the concentrations of beneficial substances superoxide dismutase, glutathione, and glutathione peroxidase were decreased compared to non-tumor tissues.

NO inhibit or promotes tumor growth depending on the concentration, duration of action, and tumor microenvironment, and shares similarities with oxygen radicals within the human body, thereby inducing oxidative and nitrosative DNA damage [40]. Consequently, this molecule can result in alterations in DNA sequence and impairment of DNA repair mechanisms [41, 42], ultimately contributing to the development of BC when acting over an extended period.

#### MiR-1949 and Rb1

MiRNAs, also known as microRNAs, are natural non-coding RNAs that primarily function by attaching to the 3’-untranslated region of target mRNAs. MiRNA Regulates many cellular activities, including proliferation, migration, differentiation, and cell apoptosis [43]. The retinoblastoma (RB) gene is the first oncogene identified to date. This protein, which is phosphorylated by nuclear processes, controls the progression of cell cycle. Numerous research studies have indicated that deactivation of the RB gene is crucial in the formation of numerous tumors, such as retinoblastoma [44].

Furthermore, the expression of miRNAs in the rat bladder was studied after SCI, revealing a significant dysregulation in miR-1949 expression **(**Fig. 2**)**. Through the utilization of TargetScan that a software used for the prediction of miRNA binding sites in mammals, the identification of the target gene miR-1949 revealed its potential involvement in the regulatory processes of Rb1. The specific regulatory role of miR-1949 in Rb1 was explored using qRT PCR, and it was observed that Rb1 expression was likely to be inhibited by miR-1949 translation. The findings suggest that miR-1949 may suppress Rb1, a target gene, potentially causing oncogenic effects. According to the above review, miR-1949 and Rb 1 may be associated with this disease, but there are few studies on its further step. We can only presumably infer that the mechanism of bladder cancer in SCI patients is related to the above, but the detailed mechanism needs further study. And perhaps researchers can continue to explore upper and downstream of molecules and related pathways and to understand how miRNAs promotes the development of bladder cancer after SCI.

## Discussion

The prevalence of BC in individuals with SCI has been documented with slight variations in the literature, which may cause some controversy. Several early studies reported a significant occurrence of SCI. While, subsequent studies have indicated a decrease in the prevalence of the disease, although it remains considerably elevated compared to the general population [17]. According to numerous studies, it has been consistently observed that individuals with SCI tend to develop BC at a relatively young age. Our statistical data indicate that the average age at which BC are first diagnosed is as low as 47 years in SCI, approximately 20 years earlier than no SCI. The significant death rate among individuals with SCI can be primarily attributed to their vulnerability to high-risk BC, specifically muscle-invasive, adenocarcinoma, and SCC. And Long-term use of indwelling catheters and repeated urinary tract infections are believed to be the contributing factors [7].

A survey among urologists indicates that the risk of recurring urinary tract infections may be reduced by Clean Intermittent Catheterization (CIC) or a combination of anticholinergic medications, aiming to decrease the number of such patients [45]. A study observing 133 individuals with SCI and neurogenic bladder symptoms [46] found that 35% of patients who underwent intermittent clean catheterization developed urinary tract infections. At the same time, the percentage was 48% for those with indwelling Foley catheters. Despite the potential benefits of CIC in reducing the likelihood of urinary tract infections and potentially lowering the occurrence of BC, its usage remains infrequent. According to a study conducted by Cameron, A. P. [47], long-term indwelling catheter usage remained unchanged for years in 71.1% of patients without transition to CIC. Therefore, to decrease the occurrence and possible fatality of BC, it is advisable to minimize the long-term use of indwelling catheters.

Screening for BC in patients with SCI is a topic of discussion, with varying opinions on whether and how it should be done. And clarifying the currently commonly used screening methods potential to identify BC early is important. Although the TERT promoter mutation and BLCA-4 assays described above may be used as early detection methods for BC, most investigators consider cystoscopy the preferred screening method. However, some authors have come to the opposite conclusion; Yang et al. [48] found in a study of SCI patients with long-term indwelling catheters (at least five years) that 156 cystoscopies were performed after SCI in 59 patients, and no malignancy was detected. In order to reduce morbidity and mortality in patients with SCI, it seems important to establish guidelines for screening BC criteria and methods.

A significant number of individuals who have sustained SCI commonly develop symptoms related to neurogenic bladder dysfunction, including urinary incontinence and retention. As a consequence, these individuals often require the prolonged use of a catheter for managing their bladder function. While this approach can meet individual requirements, it is susceptible to repeated urinary tract infections and persistent bladder inflammation. and BC primarily arises from these complexities. Ho et al. [13] conducted a comparative analysis on SCI-positive individuals who had long-term indwelling catheters. And revealed that the appearance of BC in the SCI group (0.551%) was comparable to that of the non-SCI group (0.88%). Furthermore, the two groups had no significant differences in mortality rates (SCI group: 0.23%; non-SCI group: 0.14%). These findings support the concept that BC complications in patients with SCI are primarily attributed to the prolonged use of indwelling catheters. To reduce the risk of urinary system infection and reduce the occurrence of bladder cancer, we can use clean intermittent catheterization to replace long-term indwelling catheterization, as it is more consistent with normal physiology, and no foreign body continues to stimulate the bladder [49]. Unfortunately, most studies have shown that the proportion of CIC in SCI patients has gradually decreased over time [50]. This is a problem to which specific attention should be paid when treating such patients.2.

These findings are similar to the study conducted by Ismail et al [51]. However, this study included the possible molecular mechanisms of bladder cancer in SCI patients, which may provide some help for subsequent molecular studies.

The molecular basis of BC in individuals with SCI remains unclear, and limited research is available. An examination of the literature revealed that NO significantly impacts BC progression. Chronic bladder inflammation is common among individuals with SCI who are in long-term catheterization. Neutrophils and macrophages in inflamed tissues secrete iNOS, which can facilitate the generation of substantial quantities of NO from L-arginine within the body. The harmful reaction of NO could be associated with the chemical interaction between NO and superoxide, forming peroxynitrite [52]. Peroxynitrite, a potent oxidizing agent, can harm DNA through detrimental effects on the structure of mitochondria [53, 54].Additionally, it stimulates the generation of reactive oxygen species [55, 56], leading to genetic alterations in the nucleus that may eventually lead to cancer development. Rb1, the initial oncogene discovered, functions as a crucial cell cycle modulator, exerting a significant influence on the integrity of chromosome architecture. The product of the Rb1 gene hinders the progression of the G1 to S phase in the cell cycle [57], restricting the proliferation of malignant cells, stopping cancer progression. Hence, in individuals with SCI, the miR-1949 gene might facilitate the advancement of BC by suppressing the translation of Rb1. Some studies have shown that RB 1 can be used as a predictor of bladder cancer recurrence after BCG treatment [58]. But whether bladder cancer can predict after SCI is unclear. Other oncogenic details for both molecules are currently unknown, mainly because the pathological micro-environment in the bladder after spinal cord injury (SCI) is obviously different from the normal bladder [59]. Therefore, it is necessary to explore the correlation of miRNA and Rb1 with the occurrence and development of bladder cancer after SCI as the path of pure bladder cancer related molecules may not be appropriate for bladder cancer patients after SCI. Reviewing the literature shows that there are very few molecular studies on such diseases. But this finding may help scholars continue to explore the molecular mechanism of bladder cancer in SCI patients.

## Conclusion

Patients with SCI exhibit distinct characteristics when it comes to BC, such as a significant presence of muscle-invading and non-urethral epithelial cell carcinomas, as well as an earlier onset at a younger age. To increase lifespan and decrease morbidity and mortality, it is crucial to establish screening initiatives and implement post-treatment strategies for BC in patients with SCI, as it is crucial to accurately control the treatment “site” and grasp prevention time. Before the patient develops bladder cancer. The majority of our present comprehension regarding **BC development in SCI** patients is situated within the domain of epidemiology, where there is a lack of definitive criteria outlining screening protocols for patients with BC and limited investigation into the molecular mechanisms underlying the onset of BC in patients with SCI. Thus, additional research on molecular mechanisms is necessary, in order to fundamentally understand the mechanisms by which SCI patients progress to bladder cancer and to accomplish this task, we should pay more attention to such patients and discover deeper mysteries.

However, study is limited by several factors. First, there were differences in the reporting of the included studies. Second, some of the studies were retrospective studies with risk of bias. Third, some studies have only introduced a limited number of patients, and the results are not generalisable. Fourth, during the literature screening, due to the small number of relevant literature, some reports with incomplete results were included in the studies.

### Electronic supplementary material

Below is the link to the electronic supplementary material.

Supplementary Material 1

## Data Availability

Not applicable.

## Abbreviations

BC: Bladder Cancer
SCI: Spinal Cord Injury
UC: Urothelial Carcinoma
TCC: Transitional Cell Carcinoma
SCC: Squamous Cell Carcinoma
GBD: Global Burden of Diseases
SMR: Standardized Mortality Ratio
TERT: Telomerase Reverse Transcriptase
NO: Nitric Oxide
iNOS: Inducible NO Synthase
cGMP: cyclic-Geranyl Phosphate
RB: Retinoblastoma
CIC: Clean Intermittent Catheterization
CIDC: Chronic IndwellingUurinary Catheters
N/A: Not Available

## References

[1] Lobo N, Afferi L, Moschini M (2022). Epidemiology, screening, and Prevention of bladder Cancer[J]. Eur Urol Oncol.

[2] Siegel RL, Miller KD, Fuchs HE (2022). Cancer statistics, 2022[J]. CA Cancer J Clin.

[3] Alanee S, Alvarado-Cabrero I, Murugan P (2019). Update of the International Consultation on Urological diseases on bladder cancer 2018: non-urothelial cancers of the urinary bladder[J]. World J Urol.

[4] Gui-Zhong L, Li-Bo M (2017). Bladder cancer in individuals with spinal cord injuries: a meta-analysis[J]. Spinal Cord.

[5] Bothig R, Kowald B, Fiebag K (2021). Bladder management, severity of injury and period of latency: a descriptive study on 135 patients with spinal cord injury and bladder cancer[J]. Spinal Cord.

[6] Injury G, B D T B, Spinal Cord Injury C (2019). Global, regional, and national burden of traumatic brain injury and spinal cord injury, 1990–2016: a systematic analysis for the global burden of Disease Study 2016[J]. Lancet Neurol.

[7] Phe V (2022). Bladder Cancer in neurogenic Patients[J]. World J Urol.

[8] Ali P, Lefevre C, Perrouin-Verbe B, et al. [Bladder cancer in neurogenic patients: a retrospective study of management and follow-up][J]. Progrès en Urologie; 2017. 10.1016/j.purol.2017.10.012 29174817

[9] Bothig R, Tiburtius C, Schops W (2021). Urinary bladder cancer as a late sequela of traumatic spinal cord injury[J]. Mil Med Res.

[10] Michel F, Cancrini F, Bensadoun H (2022). Incidence of bladder cancer in neuro-urological patients in France: a nationwide study[J]. World J Urol.

[11] Bothig R, Golka K, Tiburtius C (2020). Incidental bladder cancer at initial urological workup of spinal cord injury patients[J]. Spinal Cord Ser Cases.

[12] Böthig R, Kowald B, Fiebag K et al. Bladder management, severity of injury and period of latency: a descri ptive study on 135 patients with spinal cord injury and bladder cancer[J]. Spinal Cord, 59(9): 971–7. 10.1038/s41393-021-00651-3 PMC8210732 34140636

[13] Ho CH, Sung KC, Lim SW (2015). Chronic indwelling urinary catheter increase the risk of bladder Cancer, even in patients without spinal Cord Injury[J]. Med (Baltim).

[14] Ammirati E, Geretto P, Giammo A (2023). Management of complex ischial-urethral fistula in neurogenic patients performing clean intermittent self-catheterization[J]. Urologia.

[15] 35(th.) European Congress of Pathology - Abstracts[J]. Virchows Arch, 2023: 1–391. 10.1007/s00428-023-03602-w 37658196

[16] Nahm LS, Chen Y, Devivo MJ (2015). Bladder cancer mortality after spinal cord injury over 4 decades[J]. J Urol.

[17] Boethig R, Kurze I, Fiebag K (2017). Clinical characteristics of bladder cancer in patients with spinal cord injury: the experience from a single centre[J]. Int Urol Nephrol.

[18] Dobruch J, Daneshmand S, Fisch M (2016). Gender and bladder Cancer: a collaborative review of etiology, Biology, and Outcomes[J]. Eur Urol.

[19] Aveta A, Cacciapuoti C, Barone B et al. The impact of meat intake on bladder Cancer incidence: is it really a relevant risk?[J]. Cancers, 2022, 14(19). 10.3390/cancers14194775 PMC9564157 36230700

[20] Liu B, Welk B (2020). Urological malignancies in neurogenic patients[J]. Curr Opin Urol.

[21] Mühlbauer J, Klotz D, Büttner S, et al. Bladder cancer in patients with neurogenic bladder disorder: a comparative study of different etiologies[J]. World Journal of Urology; 2022. 10.1007/s00345-021-03922-z 35034168

[22] Nseyo U, Santiago-Lastra Y (2017). Long-term complications of the neurogenic Bladder[J]. Urol Clin North Am.

[23] Takahashi R, Kimoto Y, Maki T et al. Postinjury Bladder Overdistension Deteriorates the Lower Urinary Tract ‘s Storage Function in Patients with Spinal Cord Injury[J]. Urol Int, 104(7–8): 604–9. 10.1159/000508418 32594087

[24] Takahashi R, Yunoki T, Naito S, INCREASED EXCITABILITY OF BLADDER AFFERENT NEURONS IN RATS WITH SPI NAL CORD INJURY: A ROLE OF A-TYPE VOLTAGE-GATED POTASSIUM CHANNELS[J]. J Urol, 187(4S).

[25] Von Siebenthal M, Akshay A, Besic M et al. Molecular characterization of non-neurogenic and neurogenic lower urinary tract dysfunction (LUTD) in SCI-Induced and partial bladder outlet obstruction mouse Models[J]. Int J Mol Sci, 2023, 24(3). 10.3390/ijms24032451 PMC9916488 36768773

[26] Taylor AS, Newell B, Chinnaiyan AM (2022). TERT promoter mutations in Keratinizing and nonkeratinizing squamous metaplasia of the urinary Tract[J]. Eur Urol Open Sci.

[27] Hollingsworth JM, Rogers MA, Krein SL (2013). Determining the noninfectious complications of indwelling urethral catheters: a systematic review and meta-analysis[J]. Ann Intern Med.

[28] Kalisvaart JF, Katsumi HK, Ronningen LD (2010). Bladder cancer in spinal cord injury patients[J]. Spinal Cord.

[29] Alimi Q, Hascoet J, Manunta A (2018). Reliability of urinary cytology and cystoscopy for the screening and diagnosis of bladder cancer in patients with neurogenic bladder: a systematic review[J]. Neurourol Urodyn.

[30] Lee WY, Sun LM, Lin CL et al. Risk of prostate and bladder cancers in patients with spinal cord injury: a population-based cohort study[J]. Urol Oncol, 2014, 32(1): 51 e1-7. 10.1016/j.urolonc.2013.07.019 24239459

[31] Davies B, Chen JJ, Mcmurry T (2005). Efficacy of BTA stat, cytology, and survivin in bladder cancer surveillance over 5 years in patients with spinal cord injury[J]. Urology.

[32] Kinde I, Munari E, Faraj SF (2013). TERT promoter mutations occur early in urothelial neoplasia and are biomarkers of early disease and disease recurrence in urine[J]. Cancer Res.

[33] Welk B (2022). The argument against screening for bladder cancer in neuro-urological patients[J]. World J Urol.

[34] Br K, Ts N, Brenes G et al. Clinical usefulness of the novel marker BLCA-4 for the detection of bladder cancer[J]. J Urol 2000 Sep, 164(3 Pt 1):634–9. 10.1097/00005392-200009010-00004 10953114

[35] Hall CN, Reynell C, Gesslein B (2014). Capillary pericytes regulate cerebral blood flow in health and disease[J]. Nature.

[36] Picon-Pages P, Garcia-Buendia J, Munoz FJ (2019). Functions and dysfunctions of nitric oxide in brain[J]. Biochim Biophys Acta Mol Basis Dis.

[37] Rosselli M, Keller PJ, Dubey RK (1998). Role of nitric oxide in the biology, physiology and pathophysiology of reproduction[J]. Hum Reprod Update.

[38] Kröncke Kd FK, Kolb-Bachofen V (1998). Inducible nitric oxide synthase in human diseases[J]. Clin Exp Immunol.

[39] Gecit I, Eryilmaz R, Kavak S (2017). The prolidase activity, oxidative stress, and Nitric Oxide Levels of Bladder Tissues with or without Tumor in patients with bladder Cancer[J]. J Membr Biol.

[40] Sawicka ELA, Kowal P, Długosz A (2015). The role of oxidative stress in bladder cancer[J]. Postepy Hig Med Dosw (Online).

[41] Bm W, Rr. D MM (2001). Inducible nitric oxide synthase in the bladder of spinal cord injured patients with a chronic indwelling urinary catheter.[J]. J Urol.

[42] Shih YL, Hsu SY, Lai KC, et al. Allyl Isothiocyanate induces DNA damage and inhibits DNA repair-associated proteins in a human gastric cancer cells in vitro[J]. Environ Toxicol; 2023. 10.1002/tox.24020 37966020

[43] Sharma R, Malviya R (2023). miRNAs involvement in the etiology and targeted therapy of bladder cancer: Interaction between signaling pathway[J]. Precision Med Sci.

[44] Xu P, Guo F, Xiao W (2023). Generation and characterization of two induced pluripotent stem cell lines from conjunctiva of a retinoblastoma patient[J]. Stem Cell Res.

[45] Fendereski K, Hebert KJ, Matta R (2023). Variation in provider practice patterns and the Perceived need for a Shared decision-making Tool for neurogenic lower urinary tract Dysfunction[J]. Urology.

[46] Rabadi MH, Aston C (2015). Complications and urologic risks of neurogenic bladder in veterans with traumatic spinal cord injury[J]. Spinal Cord.

[47] Cameron AP, Wallner LP, Tate DG (2010). Bladder management after spinal cord injury in the United States 1972 to 2005[J]. J Urol.

[48] Yang CC, Clowers DE (1999). Screening cystoscopy in chronically catheterized spinal cord injury patients[J]. Spinal Cord.

[49] Wu SY, Jhang JF, Liu HH et al. Long-term surveillance and management of Urological complications in chronic spinal cord-injured Patients[J]. J Clin Med, 2022, 11(24). 10.3390/jcm11247307 PMC9785560 36555924

[50] Lane GI, Driscoll A, Tawfik K (2018). A cross-sectional study of the catheter management of neurogenic bladder after traumatic spinal cord injury[J]. Neurourol Urodyn.

[51] Ismail S, Karsenty G, Chartier-Kastler E (2018). Prevalence, management, and prognosis of bladder cancer in patients with neurogenic bladder: a systematic review[J]. Neurourol Urodyn.

[52] Kaneko T, Yoshioka M, Kawahara F et al. Effects of plant- and animal-based-protein meals for a day on serum nitric oxide and peroxynitrite levels in healthy young men[J]. Endocr J, 2024. 10.1507/endocrj.EJ23-0355 38220201

[53] Demiryurek AT, Cakici I, Kanzik I (1998). Peroxynitrite: a putative cytotoxin[J]. Pharmacol Toxicol.

[54] Ahmad R, Warsi MS, Abidi M (2024). Structural perturbations induced by cumulative action of methylglyoxal and peroxynitrite on human fibrinogen: an in vitro and in silico approach[J]. Spectrochim Acta Mol Biomol Spectrosc.

[55] Genovese T, Mazzon E, Mariotto S (2006). Modulation of nitric oxide homeostasis in a mouse model of spinal cord injury[J]. J Neurosurg Spine.

[56] Maggio DM, Singh A, Iorgulescu JB et al. Identifying the long-term role of Inducible Nitric oxide synthase after Contusive spinal cord Injury using a transgenic mouse Model[J]. Int J Mol Sci, 2017, 18(2). 10.3390/ijms18020245 PMC5343782 28125047

[57] Cruz-Gálvez CC, Ordaz-Favila JC, Villar-Calvo VM et al. Retinoblastoma: review and new insights[J]. Front Oncol, 12: 963780. 10.3389/fonc.2022.963780 PMC9670800 36408154

[58] Esuvaranathan K, Chiong E, Thamboo TP et al. Predictive value of p53 and pRb expression in superficial bladder canc er patients treated with BCG and interferon-alpha[J]. Cancer, 109(6): 1097–105. 10.1002/cncr.22503 17311305

[59] Wang T, Liu Y, Yuan W (2015). Identification of microRNAome in rat bladder reveals miR-1949 as a potential inducer of bladder cancer following spinal cord injury[J]. Mol Med Rep.

