# Supplementary material for: Clinical characteristics and molecular mechanisms underlying bladder cancer in individuals with spinal cord injury: a systematic review
Source: BMC Urol. 2024 May 22;24:111. doi: 10.1186/s12894-024-01457-0 (PMC11110351; doi:10.1186/s12894-024-01457-0)
Supplement: Supplementary file 1 — Supplementary Material 1 [file 12894_2024_1457_MOESM1_ESM.docx]

Supplementary Table S1. The score chart of NOS

| Year | Author | Slelection | | | | Comparability | Outcome | | | Points | Quality of Studies |
| --- | --- | --- | --- | --- | --- | --- | --- | --- | --- | --- | --- |
|  |  | 1 | 2 | 3 | 4 | 5 | 6 | 7 | 8 |  |  |
| 2015 | Laura S | * | * | * | * | * | * | * | * | 8 | High |
| 2022 | Floriane M | * | * | * | * | ** | * | * | — | 8 | High |
| 2020 | Ralf Böthig | * | — | * | * | * | * | * | — | 6 | High |
| 2017 | Gui-Zhong, L | — | — | * | * | — | * | * | — | 4 | Intermediate |
| 2015 | Ho, C.H | * | * | * | * | ** | * | * | * | 9 | High |
| 2021 | Ralf Böthig | * | * | * | * | ** | * | * | * | 9 | High |
| 2021 | Ammirati Enrico | * | — | — | — | — | * | — | — | 2 | Low |
| 2023 | Hidalgo Romero A | * | — | — | — | — | * | — | — | 2 | Low |
